# Mediterranean Diet Adherence in Community-Dwelling Older Adults in Spain: Social Determinants Related to the Family

**DOI:** 10.3390/nu14235141

**Published:** 2022-12-02

**Authors:** Rut Navarro-Martínez, Mayra Alejandra Mafla-España, Omar Cauli

**Affiliations:** 1 Department of Nursing, University of Valencia, 46010 Valencia, Spain; 2 Frailty Research Organized Group (FROG), University of Valencia, 46010 Valencia, Spain; 3 Department of Hematology, Hospital General Universitario, 46014 Valencia, Spain

**Keywords:** Mediterranean diet, family, social determinants, public health

## Abstract

The Mediterranean diet (MD), a well-established quality diet model, and regular physical activity are associated with reducing the appearance or progression of several chronic diseases and reducing morbidity and mortality. However, reduction of these goals, adherence to the MD, and regular physical activity occur at all ages, including older individuals in Mediterranean countries such as Spain, where at least adherence to the MD is culturally rooted. Objective: To evaluate the degree of adherence to the MD and physical activity in older individuals. Methods: The sample comprises 679 older adults aged 60 and over who attended activities in municipal centers for older adults in Valencia. Adherence to the Mediterranean diet, frequency of physical activity, and anthropometric assessment were used. Results: High adherence (score ≥ 9) to MD was observed only in 23.7% of the study sample. Smoking habits or having meals in fast-food restaurants on a weekly basis were significantly (*p* < 0.05) associated with lower MD adherence. Age, BMI, marital status, and physical activity were not significantly associated with MD adherence. Physical activity was significantly (*p* < 0.05) lower in individuals who were divorced or widow/ers and in those taking care of their grandchildren several times a week. Conclusions: Adherence to the MD in a big Spanish city is low among older individuals. Socio-family factors seem to play a role. Public health and governmental strategies should reinforce adherence to the MD among older individuals as a gold standard for nutrition.

## 1. Introduction

Various epidemiological studies have analyzed the relationship between dietary habits and the onset of chronic diseases [1,2,3]. Adopting unhealthy lifestyles with poor dietary habits with little or no physical activity contributes to an increased risk of obesity and multiple chronic degenerative diseases [4]. According to the World Health Organization (WHO) [5], nearly 30% of the population in Europe is obese. This demonstrates that diet is a key factor in developing these pathologies, which has a greater impact on the elderly population [6]. In fact, from age 50 onwards, different body and lifestyle changes begin that can considerably affect nutritional status and, thus, health [7]. In this regard, older adults with a better-quality diet have a lower risk of suffering from chronic diseases [8,9]. In specific terms, the Mediterranean Diet (MD) model as a quality diet is associated with reduced cardiovascular risk and the prevention of chronic diseases and some types of cancer [10,11,12,13]. According to the original definition [14], the MD is characterized by a high intake of vegetables and fruits and a high consumption of vegetables, legumes, whole-grain cereals, fruits, nuts, dried fruits, and olive oil (as the main source of fat in the diet). It is also characterized by moderate consumption of fish, and dairy products, low consumption of meat and meat by-products, and regular but moderate consumption of wine during meals [15]. However, the Mediterranean diet cannot only be considered a healthy eating pattern but instead encompasses a healthy lifestyle, with respect for traditions and moderate physical exercise [16,17]. However, industrialization, urbanization, economic development, and globalization of the food market are changing eating habits and lifestyles at all ages [18,19,20,21]. This is all leading to the adoption of sedentary lifestyles (which consequently reduce energy expenditure) and the choice of foods of poorer nutritional quality (such as snacks and refined, processed, and high-fat foods) [22,23].

At the international level, the study on the elderly population living in Mediterranean areas (MEDIS) concludes that good adherence to the Mediterranean diet is associated with a decrease in body weight and blood pressure [24]. Similar results were obtained in three European studies at the European level: The Healthy Ageing Longitudinal Study in Europe (HALE) [25,26], the European Prospective Investigation into Cancer and Nutrition (EPIC) [27]; and a meta-analysis conducted on prospective cohort studies, the results of which suggest that good adherence to the MD is associated with lower overall mortality, and more specifically with a greater reduction in coronary reduction [10]. Other studies conclude that MD is associated with a reduction in mortality and an improvement in the quality of life, despite the fact that adherence to the Mediterranean Diet is declining in the Spanish population, with undesirable effects on health. It is, therefore, necessary to carry out campaigns to promote healthy eating among this population group [23,28,29,30]. In the last years, globalization, industrialization, a rapid increase in fast-food restaurants, and advances in the food industry have transformed the traditional MD of most Mediterranean regions into a more global and ‘Western’ or industrial dietary patterns typically described as well-implemented in child/adolescent population [31]. These diet changes, combined with a more sedentary lifestyle, are major contributors to the increase in non-communicable diseases. They could have also reached the dietary habits of older individuals in Mediterranean countries, obesity and cardiovascular disease, and some type of cancer epidemic of the last decades [1,2,3]. In addition, social family changes occurring in Spanish society and other countries showed a growing number of grandparents are now taking on the parenting role for their grandchildren, thus foregoing the traditional grandparent/grandchild relationship [32,33,34]. This often means taking on responsibility for the day-to-day maintenance of a home, schedules, meals, homework, and play dates [35,36,37]. This role could influence the time available for doing physical activity or good adherence to MD. This study aims to evaluate community-dwelling older individuals and the relationship between adherence to Mediterranean Diet, body mass index, and physical activity and to address the role of sociodemographic factors.

## 2. Methods

### 2.1. Population and Sample Size Calculation

The sample size was 679 participants, which was considered sufficient for the target population of older adults attending activities in Senior Citizens’ Centers in Valencia (Spain). In order to carry out the study, after consulting the recent work on the prevalence of low adherence to MD in older individuals in Spain, it was determined that this variable ranged from 35% to 40% [38,39]. Therefore, admitting a degree of error in the estimate of 5% and with a confidence interval of 95%, a sample size of 410 subjects randomly selected will suffice. A replacement rate of 10% was anticipated for those questionnaires lacking some information. The final sample was 679 individuals, which exceeds 269 cases, which allows us to make an acceptable estimate of the prevalence of low adherence to the MD diet. The sample size calculations were done with the EPIDAT 4.2 software. The data collection took place between March 2021 and May 2022.

### 2.2. Ethical Considerations

This study received approval from the ethical committee of the University of Valencia (approval reference 1577979, 4 March 2021). The respondents provided written informed consent before completing the questionnaire, and their confidentiality was protected. The collected data were solely used for scientific and research purposes.

### 2.3. Study Tool

An anonymous survey was distributed among the participants. The structure of the survey included (1) sociodemographic data, (2) the validated Mediterranean Diet Adherence questionnaire [40], and the International Physical Activity Questionnaire (IPAQ) [41,42], both of which were validated in Spanish.

### 2.4. Mediterranean Diet Adherence Questionnaire

A specific short questionnaire of fourteen items was validated for the Spanish population and used by the Prevention with Mediterranean Diet (PREDIMED) group to determine the degree of adherence to the Mediterranean diet. To obtain the score, a value of +1 was assigned to each item with a positive connotation with respect to the MD and −1 when the items had a negative connotation. Based on the sum of the values obtained for the 14 items, the score of adherence was classified as an overall score of <9 points representing participants with low adherence, while an overall score of ≥9 points was used to identify participants with high adherence to the MD [30].

### 2.5. Physical Activity Assessment

The short self-administered IPAQ [42] was used to assess physical activity. The IPAQ focuses on the amount of physical activity performed over the past seven-day period. The IPAQ includes questions about the time spent engaging in vigorous physical activity, moderate physical activity, and walking. Within these domains, participants are asked to consider all types of physical activity, including activities performed during leisure time, domestic and gardening, work-related, and transport-related activities. The data obtained from the IPAQ was used to estimate the total amount of physical activity completed in a seven-day period by weighting the reported minutes per week in each domain by a MET (metabolic equivalent) energy expenditure estimate. The weighted MET minutes per week were then calculated by multiplying the duration (minutes), frequency (days), and MET intensity and then summing across the three domains, namely vigorous, moderate, and walking, to produce a weighted estimate of total physical activity per week (MET.min.wk-1) [42].

### 2.6. Anthropometric Assessment

Height and weight were measured, and body mass index (BMI) was computed as kg/m^2^. BMI was categorized using the WHO classification, with BMI < 18.5 as underweight, 18.5–24.9 as normal weight, 25–29.9 as overweight, and >30 as obesity. For participants aged <65 years old [43], BMI was categorized according to the guidelines for older (aged 65 and over) individuals in the Spanish population as underweight (BMI under 22.0 kg/m^2^), normal weight (BMI between 22.0 and 26.9 kg/m^2^), overweight (BMI between 27.0 and 29.9 kg/m^2^) and obesity (BMI over 30.0 kg/m^2^) [7].

### 2.7. Statistical Analysis

Descriptive statistics were calculated for each variable. The results are presented as means and frequencies. A descriptive analysis of the frequency of consumption of the 14 items of the MD adherence questionnaire was performed to calculate the percentage of the population complying with the nutritional recommendations. The *p*-value was calculated using the Mann-Whitney U and Kruskal-Wallis non-parametric tests since the distribution of the continuous variables was not normal, and the χ2 analysis for categorical variables was used to verify the existence of statistically significant differences. An MD score of ≥9 was used to determine their high adherence to MD, according to other research [30]. In order to estimate the magnitude of the significant changes (*p* < 0.05) between the two experimental groups, we calculated Cohen’s d effect size. Logistic regression analyses were used to predict the probabilities of the different social and health factors associated with good adherence to MD. The result expressed as odds ratios (ORs), 95% confidence intervals (95% CIs), and statistical significance (*p*) were estimated for each variable significantly associated with the bivariate analyses. The SPSS version 28.0 statistical package was used to obtain the results.

## 3. Results

### 3.1. Sociodemographic Factors, Body Mass Index, and Smoking Habits

We collected 679 questionnaires from individuals aged 60 and over (mean age 73.5 ± 0.2 years (range 60–94)), all of whom lived in Valencia (Spain), of whom 13.8% were men (*n* = 94) and 86.2% were women (*n* = 585). Marital status and the number of sons/daughters cohabiting in the same home are reported in Table 1. We also ask participants about their role in caring for and parenting their grandchildren, if any (e.g., accompanying them to and from school, preparing meals for children, helping with extra-curricular activities, etc.), including the number of days in a week where they are occupied with those tasks. Data related to smoking and altered BMI index are also shown in Table 1.

### 3.2. Adherence to MD

Analysis of adherence to MD revealed a mean score of 7.5 ± 0.6 (3–13 range). The MD score of ≥9 was used to determine high adherence to MD. High adherence was observed in 161 individuals, 23.7% of the study sample. Scores of 7 and 8 were the most frequent in the study sample, accounting for 25.0% and 25.2%, respectively. Analyzing by quartiles, the highest quartile of adherence to MD was a score of 8. Based on the score of 8, 48.2% (*n* = 327) of the study sample had 8 or more. Categorization by the lowest quartile of MD adherence (score ≤ 6) revealed that 26.5 % (*n* = 180) had poor adherence to MD. The recommended pattern for each item was a score of 1, whereas those below were scored 0, as previously validated in the Pedi-Med stud [44].

The frequency of individuals following the recommended pattern for each of the 14 items in the adherence questionnaire is presented in Table 2. The items with the lowest adherence to the recommendation were item 8—“drinking wine” (5.4%), item 9—“servings of legumes” (17.9%), item 14—“sofrito seasoning” (17.5%), and item 13—“white meat consumption instead of red meat” (23.3%). 12.2% (*n* = 83) of the participants reported having lunch or dinner in a fast food restaurant once or more a week.

**Figure 1:**
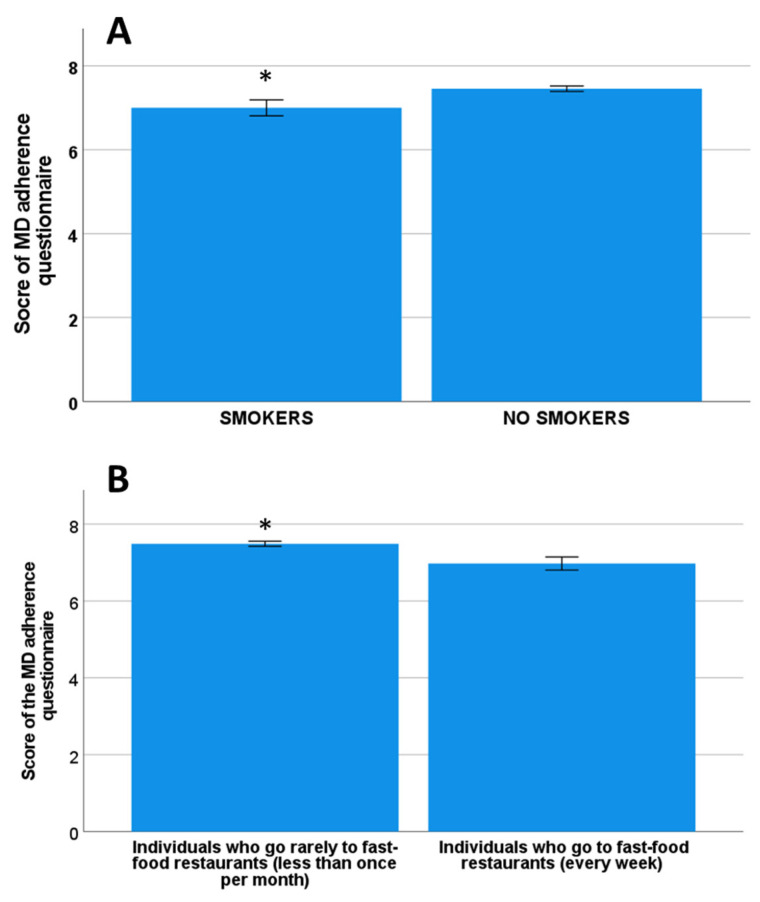
Mean score of MD adherence questionnaire in smokers and non-smokers (**A**) and those individuals having or not having main meals in fast-food restaurants (**B**). * *p* < 0.05.

No significant differences were observed between the recommended adherence to MD (based on 9 score cut-off) and gender (*p* = 0.89), age categorized as younger than 75 years old and ≥75 years old (*p* = 0.75), marital status (*p* = 0.84), level of education (*p* = 0.09), having sons/daughters, grandchildren living in the same house (*p* = 0.44), taking care of grandchildren (*p* = 0.17) or taking grandchildren to school (*p* = 0.33), or BMI categories (*p* = 0.46), or being obese versus no obese (*p* = 0.78), or adding overweight individuals (as overweight/obesity group) (*p* = 0.80). A significant association was found with the smoking habit (*p* = 0.028), having the smokers a significantly lower adherence to MD compared to non-smokers (7.0 ± 1.9 and 7.5 ± 0.1 respectively, *p* = 0.013, Cohen’s d = 0.3) (Figure 1A). Individuals that have lunch or dinner once (or more times) in a week in fast-food restaurants have significantly lower adherence to MD compared to individuals that never or almost never go to fast-food restaurants (6.98 ± 0.17 and 7.49 ± 0.07 respectively, *p* = 0.02, Cohen’s d = 0.3) (Figure 1B). When we dichotomized the score of the adherence to MD by the recommended adherence (score ≥ 9) and below (score < 9), we included the variables with significant differences in bivariate analysis in the model. The logistic regression analysis showed that the smoking habit is significantly (*p* = 0.03) associated with a lower score of adherence to MD with an OR of 2.78 (CI95: 1.081–7.142).

### 3.3. Level of Physical Activity

**Figure 2:**
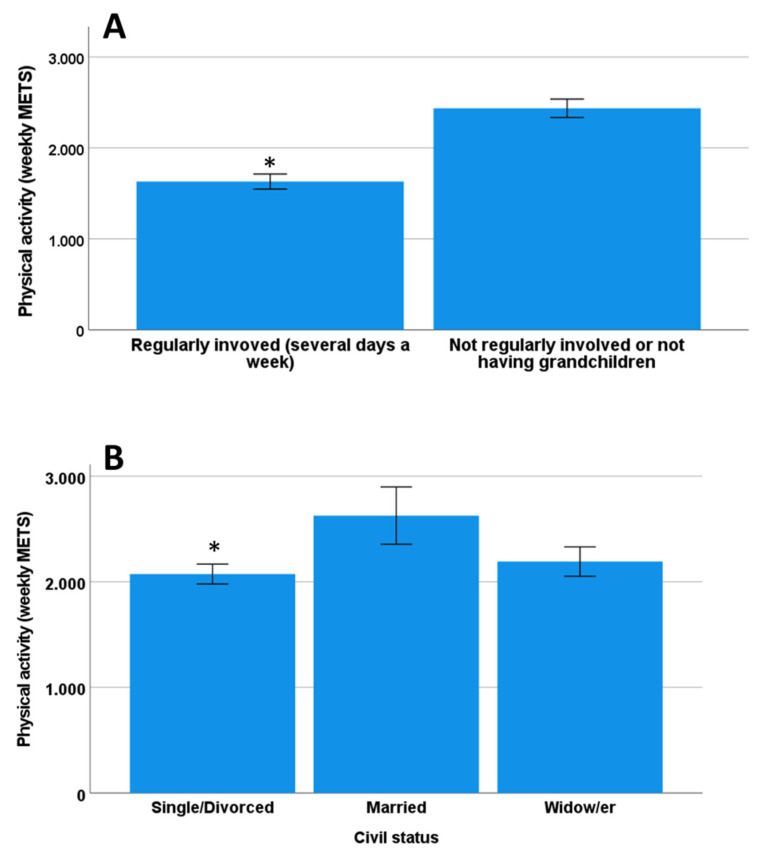
Physical activity (weekly METS) in participants involved or not in the care and parenting tasks (**A**) in participants according to their civil status (**B**). * *p* < 0.05.

The mean metabolic equivalents of task (METS) of physical activity on a weekly basis was 2165.9 ± 74.0. 8.9% (*n* = 59) of the study sample referred to intense physical activity, and 70.0% (*n* = 475) referred to moderate physical activity. 5.6% (*n* = 38) referred to no physical activity with 0 METS. A large percentage (80.6%) of the study sample reported that their apartment was in a building with a lift or that they lived in a home on the ground floor. There was a significant direct correlation between the METS obtained doing intense or moderate physical activity or walking (*p* < 0.01 in all comparisons, Spearman or Pearson correlation analysis). The most significant correlation with total METS was observed with walking (rho = 0.816, *p* < 0.001), suggesting the main contributor to physical activity is walking time. The total METS was significantly different between categories of some sociodemographic factor, e.g., those taking care of grandchildren had fewer METS compared to those who did not (*p* < 0.001, Cohen’s d = 0.42) (Figure 2A) or those taking them to school had significantly fewer METS than those who did not (*p* < 0.001, Cohen’s d = 0.33), and marital status (*p* = 0.03, Cohen’s d = 0.23 those who were married had significantly higher METS than those who were widow/ers and single/divorced) (Figure 2B). The other sociodemographic factors and healthy habits did not show significant differences with the weekly rate of physical activity (total METS).

### 3.4. Relationship between Adherence to MD and Healthy Habits

There was no significant correlation between adherence scores to MD and total weekly METS (rho = 0.013, *p* = 0.73), METS obtained by doing intense physical activity (rho = 0.028, *p* = 0.47), moderate physical activity (rho = 0.017, *p* = 0.66) and walking (rho = −0.019, *p* = 0.62). When we dichotomized the total METS by quartiles, the individuals with the highest quartile showed no significant association with categorical MD scores based on the score ≥ 9 (*p* = 0.81) for either the highest quartile in MD adherence (score ≥ 8) (*p* = 0.90) or the lowest quartile (score ≤ 6) (*p* = 0.94). The correlations between the MD adherence score and total METS remain non-significant (*p* > 0.05 in all cases), also considering the variables together (*p* = 0.74) or alone (data not shown) (smoking, weekly fast food restaurant visits, and lifts to enter and leave home) which have a significant effect per se on the adherence score to MD as shown before. The correlations between the MD adherence score and total METS remain non-significant (*p* > 0.05 in all cases), also considering the variables together (*p* = 0.56) and alone (data not shown) (taking care of grandchildren, taking them to school, marital status) which have a significant effect per se on the adherence score to MD as shown above. There was no significant correlation between adherence to MD and BMI (rho = −0.042, *p* = 0.28). Categorizing the BMI into four categories (underweight, normal weight, overweight, and obesity) showed no significant difference in MD adherence scores (*p* = 0.65, Kruskal-Wallis test).

## 4. Discussion

A healthy diet and lifestyle, such as regular physical activity, may help prevent the onset of age-related diseases and positively affect their evolution, thereby promoting the Healthy Aging process, a concept recently coined to describe the disease-free aging process. Regarding diet, the Mediterranean diet Model has proven useful for supporting healthy aging, and health policies to promote this lifestyle seem to be the best approach for achieving this target [45,46].

In our study, the data show that in a population in a Spanish city, where the Mediterranean diet should, in theory, be culturally established, only 23.7% of the population studied had high adherence to the MD (score of ≥9). Unfortunately, these data highlight the results of [47] on the decline in MD adherence in classically Mediterranean countries such as Spain, which are moving away from the traditional MD pattern, and adopting a less varied and more “westernized” diet typical of countries in northern Europe and the United States [48,49]. In the current sample, this distancing from the traditional Mediterranean model is observed mainly in their high levels of consumption of commercial pastries, carbonated and sugary drinks, and red or processed meats compared to white meats, which are not typical foods of the Mediterranean region. Likewise, the intake of some basic foods in the MD, such as vegetables, cereals, legumes, and fish and shellfish, was lower than expected. This giving up of traditional eating habits may, in large part, be due to a progressive homogenization of eating behavior as a result of the widespread dissemination of Western culture, as well as globalization in the production and consumption of food [8]. Furthermore, unhealthy foods also have larger advertising budgets, and this partially makes decisions aimed at healthier dietary patterns more difficult [4]. Likewise, several barriers can potentially limit adherence to MD, such as a long preparation time for some of the foods included in MD, such as legumes, which should be consumed frequently. Only 17.9% of our sample reported consuming legumes three or more times a week, far below the Spanish average consumption of 43% [38]. Food choice is also related to hedonic aspects centered on taste [50]. Given that high-calorie foods show high taste expectancy, it could justify a higher consumption of commercial pastries, carbonated and sugary drinks, and red or processed meats by our participants, with a consequent loss of MD adherence [51]. Only 5.4% of the participants reported drinking wine regularly with meals. Given that most study participants were women, low wine consumption may also be related to different habits between genders [52]. Most of the literature agrees that light to moderate wine intake has beneficial effects on chronic non-communicable diseases, such as hypertension, cancer, dyslipidemia, and dementia. Still, no definitive recommendations can be made on a specific dose intake that can benefit most diseases [53,54]. However, It should be further considered that other hallmarks of the Mediterranean diet are the richness in virgin olive oil, fruits, grains, and vegetables, which are also good sources of polyphenols and other phytochemicals, and lack the risks of wine [55].

Previous studies have suggested that people from lower socio-economic and educational levels generally consume fruits and vegetables less often and foods rich in sugars and animal fats more frequently [20]. However, in the present study, despite the large percentage of individuals with only primary education, we found no significant differences in the percentage of adherence to MD according to educational level, although a tendency was observed towards higher adherence among individuals with a higher educational level. Other studies in Spain did not find any association either [29]. However, it should be noted that ignorance of the diet-disease link can lead individuals to ignore the risks of excessive consumption of certain types of food for their health and to fail to see the need to increase the number of fruits and vegetables they consume. In addition, healthy food may be perceived as more expensive compared to more energy-dense food groups, and this higher cost of food may pose a barrier to adherence to MD [51,56]. Although, as a limitation of our study, we do not have the income level of our participants, in Spain, with data from 2021, the average annual income of people over 65 years of age barely exceeds 14,000 euros per year [57,58] which is lower than that of the elderly in our neighboring countries. This medium-low purchasing power of the Spanish elderly could make it difficult for them to access some of the foods necessary for a healthy and balanced diet, which could lead them away from the traditional pattern of MD [15].

Our data reflects slow but steady growth in fast food consumption among older people. Indeed, around 12% of the participants in our study reported having lunch or dinner in fast-food restaurants at least once a week. It is essential to contextualize these new eating patterns within the revolutionary era we are experiencing, in which advertising, competitive prices, and the significant appeal of fast food chains for the grandchildren of the elderly, in addition to encouraging the consumption of this kind of food, has led people in this age group to replace traditional a la carte restaurants with simpler meals, such as fast food and self-service [59,60]. We also observed that this percentage was significantly higher among single older people, which could be explained by the fact that people living in a family are more likely to share food prepared at home [61,62,63]. Similarly, we observed that eating fast food in restaurants was accompanied by lower adherence to the MD. As a result, contrary to what might be expected, intake is not compensated with an intake of other healthier foods, such as fruits or vegetables. Still, on the contrary, the quality of the diet tends to deteriorate, with consequent risks.

As regards the relationship between adherence to the MD and healthy behaviors, we found that smokers had lower adherence to the MD than non-smokers. These results may be due to the fact that smoking is a habit that could associate with other less healthy behaviors and, consequently, a selection of less healthy foods [19]. These data confirm previous research that reported a less healthy dietary profile among smokers, with a higher intake of fats (mainly saturated fatty acids) and lower consumption of fruits, nuts, and fiber. Similarly, a recent study conducted in a cohort of 5293 US adults shows a direct relationship between smoking and dietary energy density [64]. These dietary imbalances in smokers can aggravate the harmful effect of tobacco on cancer and the risk of cardiovascular diseases, so it would be advisable to implement dietary interventions in smokers so that they adopt good eating habits and, to a certain extent, mitigate the effect of smoking on their health.

Regarding the level of physical activity in this study, the participants showed an average energy expenditure of physical activity of 2165 METS·min/week, which is globally higher than the recommendations of the Centers for Disease Control and Prevention (CDC) and the Organization World Health Organization, which recommend a total energy expenditure of 600 METS min/week [65]. This mean value is also higher than that observed in the international validation of the IPAQ Short version, which established an average value in adults over 65 years of age of 1581 METS min/week [66]. Considering that healthy lifestyle variables tend to cluster together, the practice of physical exercise has been associated with greater adherence to the MD [14,29,67]. However, it is surprising that in our sample, which had a physically active lifestyle, a higher energy expenditure due to physical activity did not imply a better adherence to MD. Several hypotheses could explain this finding; first, the dual profile that the elderly in our study may have, as regardless of compliance with the recommended values for physical activity, they may present sedentary behaviors such as spending more than 5 h a day in front of a screen, a popular activity among older adults [22]. Second, according to “the paradox of physical activity”, it is possible that rather than the amount of energy expended, the nature of the activities carried out has the most influence on a healthy lifestyle [68]. In our study, where the participants are mostly women, gender can act as an important discriminatory factor in the type of activities carried out. Based on the above, unlike men, women dedicate significantly more effort to domestic activities, especially household ones, than outdoor sports and leisure activities.

On the other hand, a social factor such as caring for grandchildren several times a week was associated with less physical activity. It has been suggested that grandparents who take on intensive parenting roles are often among the most disadvantaged and in the poorest health [66]. Although the extent to which these adverse outcomes are due to the impact of caring for grandchildren or to the advantages or disadvantages accumulated over the course of the individual’s life is unknown, in our study, the gender difference is once again an important aspect for understanding lower levels of self-management of health in grandparent caregivers. Accordingly, while grandfathers involved in the care of their grandchildren tend to play the roles of playmates, caregiver grandmothers fulfill more intense responsibilities, such as feeding, bathing, and dressing the grandchildren [69]. Grandfathers may have more time and energy to manage health-related behaviors, including increased physical activity. Finally, a higher level of energy expenditure in physical activity was observed among married people. Although the results of previous studies on physical activity among people of different marital statuses are inconclusive [69], interpersonal relationships between the members of a couple can influence their level of physical activity by providing social support and influencing the other person’s behavior [70]. The study has limitations that must be considered when interpreting its findings, such as the study’s cross-sectional design, which prevents the establishment of a causal relationship. Repeated measurements of adherence over time would provide a more valid and reliable estimate of MD adherence. Second, the dietary and physical activity data are based on self-reports, leading to recall and reporting bias. Another limitation of this study concerns the lack of information related to the participant’s income level, which could partially modulate the strengths of these associations reported in the study. Finally, the spatial limitation of the inhabitants of a single big city means that the sample could not be representative of those living in non-urban areas. Despite these limitations, the results of the study highlight a serious public health concern about the high rate of low adherence to MD (in contrast with good physical activity level) in community-dwelling older individuals and suggest the need for health education programs and social support to achieve better adherence.

## Figures and Tables

**Table 1:** Characteristics of the study sample.

Variable	Frequency over the Sample (%)
Gender	86.2% females, 13.8% males
Individuals with smoking habit	7.4%
Civil status	Married 57.5% Single/Divorced 10.0% Widow/er 32.5%
BMI classification	Underweight 7.7% Normal weight 47.1% Overweight 23.1% Obesity 22.1%
Percentage of older individuals living with their sons/daughters	18%
Individuals taking care of their grandchildren on a regular basis (3 times/per week)	32.7%

Continuous variables are expressed as mean ± standard error of the mean (SEM); the categorical variables are expressed as frequency (percentage) over the total sample.

**Table 2:** Percentage of individuals that fulfilled the recommended frequency of consumption (on a weekly basis) of foods included in the “MD adherence questionnaire”.

Items of the Questionnaire Corresponding to Good Adherence to MD	% of Individuals over the Total Sample Who Fulfilled the Recommended Pattern of Consumption of Each Food
Use olive oil as the principal source of fat for cooking.	96.6%
≥4 tablespoons oil/day	86.6%
≥2 servings vegetables/day	26.8%
≥3 servings fruit/day	56.6%
<1 serving red meat, hamburgers, sausage/day	56.1%
<1 serving butter, margarine, cream/day	83.8%
<1 serving carbonated sweetened beverages/day	87.6%
≥7 glasses of wine/week	5.4%
≥3 servings legumes/week	17.9%
≥3 servings fish- seafood/week	28.1%
<2 servings commercial pastries/week	90.3%
≥3 servings nuts/week	65.7%
Prefers white meat/red meat	23.3%
≥2 times/week homemade tomato sauce	17.5%
Individuals with good adherence to MD based on proposed cut-oof value (Total score ≥ 9 points) [28]	23.7%

## Data Availability

The data presented in this study are available on request from the corresponding author.
